# The role of local context for managers’ strategies when adapting to the COVID-19 pandemic in Norwegian homecare services: a multiple case study

**DOI:** 10.1186/s12913-023-09444-1

**Published:** 2023-05-16

**Authors:** Camilla Seljemo, Siri Wiig, Olav Røise, Eline Ree

**Affiliations:** 1 SHARE – Centre for Resilience in Healthcare, Faculty of Health Sciences, University of Stavanger, Stavanger, Norway; 2 Institute of Clinical Medicine, University of Oslo, Oslo, Norway; 3 Division of Orthopedic Surgery, Oslo University Hospital, Oslo, Norway

**Keywords:** Managers, Homecare services, Local context, Adaptation, Strategies, Resilience, Pandemic

## Abstract

**Background:**

The COVID-19 pandemic had a major impact on healthcare systems around the world, and lack of resources, lack of adequate preparedness and infection control equipment have been highlighted as common challenges. Healthcare managers’ capacity to adapt to the challenges brought by the COVID-19 pandemic is crucial to ensure safe and high-quality care during a crisis. There is a lack of research on how these adaptations are made at different levels of the homecare services system and how the local context influences the managerial strategies applied in response to a healthcare crisis. This study explores the role of local context for managers’ experiences and strategies in homecare services during the COVID-19 pandemic.

**Methods:**

A qualitative multiple case study in four municipalities with different geographic locations (centralized and decentralized) across Norway. A review of contingency plans was performed, and 21 managers were interviewed individually during the period March to September 2021. All interviews were conducted digitally using a semi-structured interview guide, and data was subjected to inductive thematic analysis.

**Results:**

The analysis revealed variations in managers’ strategies related to the size and geographical location of the homecare services. The opportunities to apply different strategies varied among the municipalities. To ensure adequate staffing, managers collaborated, reorganized, and reallocated resources within their local health system. New guidelines, routines and infection control measures were developed and implemented in the absence of adequate preparedness plans and modified according to the local context. Supportive and present leadership in addition to collaboration and coordination across national, regional, and local levels were highlighted as key factors in all municipalities.

**Conclusion:**

Managers who designed new and adaptive strategies to respond to the COVID-19 pandemic were central in ensuring high-quality Norwegian homecare services. To ensure transferability, national guidelines and measures must be context-dependent or -sensitive and must accommodate flexibility at all levels in a local healthcare service system.

## Background

Pandemics have been identified as one of the crisis scenarios with highest probability of occurring and with the highest consequences in Norway [1]. When the COVID-19 pandemic hit Norway in March 2020, the strongest measures in peace time were implemented by the Norwegian government resulting in a national lockdown. Preparedness plans were immediately implemented, but this unknown virus proved them insufficient [2]. Despite the lack of sufficient preparedness plans, Norway was one of the countries in Europe with the lowest mortality rate, burden of restrictions and the lowest reduction within economic activity [2]. To reduce the impact of a similar crisis like this pandemic, a healthcare system needs to treat critically ill patients while providing essential health services and safe and high-quality care. Homecare services provide healthcare services in patients’ homes and have a key role in preventing the spread of contagion [2]. In several contexts, infected patients who were considered stable were treated in their homes instead of in intensive care units [3, 4]. This has forced homecare managers to adapt to the COVID-19 pandemic (e.g., infection control measures, resource pressure, treatment of patients with complex medical conditions) and to prepare for potential outbreaks in homecare [5]. There is a need to explore how managers in homecare services experienced the pandemic, because providing at-home healthcare services requires creative approaches and solutions [6].

### Managers’ role in resilience

Healthcare managers’ ability to adapt and respond quickly to changing circumstances is an important factor in ensuring high-quality services during a crisis [7–10]. Healthcare managers are constantly coping with uncertainty and a changing environment [11]. Their responsibilities have only increased during the COVID-19 pandemic as a result of government-imposed infection control measures [2, 9]. The ability to manage uncertainty and challenges, maintain normal operations and recover from disruptions and unexpected events like pandemics, aligns with Wiig et al.’s (12, p.6) definition of resilience: “the capacity to adapt to challenges and changes at different system levels, to maintain high quality care.” Adaptation is a crucial element in resilience [6, 12–15] and occurs when standardized routines require new strategies and innovative solutions [16, 17]. Managers strategies supporting resilient performance has been defined as strategies applied to “engage people in collaborative and coordinated processes that adapt, enhance or reorganize system functioning, promoting possibilities of learning, growth, development and recovery of the healthcare system to maintain quality care” [18].

Healthcare managers are stated to support resilience through their strategies to adapt, adjust, anticipate, monitor, and make sense of new situations, challenges, or disruptions [18]. Managers adapt by realigning capacity and demands at all system levels, but strategies applied to one level might influence the ability to perform resiliently at another [14, 19] as the interaction of strategies among levels plays a significant role for improving quality of services [20]. Macrae’s framework describes how organisational activity unfolds at different moments of resilience (situated, structural and systemic). Situated resilience occurs rapidly at front-line as a response to a disruptive event. Structural resilience occurs as a response to previous disruptions in front-line and unfold over months to decades. Systemic resilience involves reconfiguration of a system as a response to disruptions and occurs over a longer period of time as in decades [16]. For example, front-line experiences based on restructuring practices can make it possible to reorganize homecare services to promote safety [16]. Managers at different levels hold responsibilities that require coordinated response and decisions for implementing immediate and long-term quality improvement measures.

Ree et al. [9] have explored nursing home and homecare managers’ strategies when responding to the COVID-19 pandemic. The strategies reported were related to situational adaptation ensuring adequate resources, education, training, and ways of conveying information in addition to promoting collaboration and cohesion. However, their study explored strategies applied close to the front-line within a single Norwegian local context.

### The role of contextual factors

Contextual factors such as geographical location are important for the experiences, management, and consequences of the COVID-19 pandemic [21, 22]. The size of a municipality, its geographical location, and cross-level collaboration and coordination have been cited by healthcare managers working with quality and safety efforts in Norwegian municipalities [23]. When a healthcare system adopts and implements macro-level guidelines and measures, they do not always fit into different contexts [15]. For example, rural and decentralized municipalities might be more vulnerable due to their limited access to healthcare services and difficulties recruiting a workforce. It is also reasonable to believe that contagion will spread more rapidly in densely populated centralized urban areas than in rural areas. Such differences require the modification of plans and strategies. This raises the legal question of whether or not there should be a one-size-fits-all strategy [24].

Based on previous literature and knowledge, there is a need for more research to understand how resilience is interlinked within and across system levels [19, 25–27]. Furthermore, there is a lack of knowledge of how crisis management and adaptation is interwoven with contextual factors [10].

### Aim and research questions

The aim of this study was to explore experiences, challenges and managerial strategies applied at different system levels (micro-meso) to manage the COVID-19 pandemic in Norwegian homecare services. We also explore how local context influences strategic responses to the pandemic in Norwegian municipalities offering homecare services. Two research questions guided the study: (1) What strategies did managers apply when adapting to challenges to ensure high-quality and safe homecare services during the COVID-19 pandemic? (2) How did the local context influence managers’ experiences and strategies when adapting to the COVID-19 pandemic?

By exploring homecare services with different geographical locations in Norway, this study will deepen the understanding of managers’ role in resilience. It will also assist in developing practical recommendations for handling future pandemics and planned changes in healthcare.

## Methods

### Design

A qualitative multiple embedded case study design was conducted [28] as it incorporates two levels of analyses – infection control doctors, top-level and middle-managers at meso level and front-line managers at micro level in four municipalities. This design allows several multi-level cases to be explored within the same context.

### Norwegian geography and demography

Norway is a finger-shaped country with many plateaus, mountains, a long coastline, and large distances between labor markets. Its population is centralized, and emigration has had a pronounced effect on the northern part of the country. Although increasing, Norway’s population is only about 5.4 million [29]. In Norway, centralization is associated with urbanization and ranked by Statistics Norway (SSB) based on the population’s access to services. The most centralized municipalities are located in the south-eastern part of Norway around the capital city of Oslo. In the northern part of Norway, municipalities are scattered, so they are considered decentralized due to their small populations. However, as much as 80% of the Norwegian population lives in densely population areas.

### Study context

The municipalities are responsible for primary healthcare and can organize their services in accordance with national laws and guidelines [30]. Primary healthcare, which includes nursing homes and homecare services, is the basic level of health services in the Norwegian health system. Homecare services include all healthcare services provided by healthcare professionals in the recipient’s home, such as home nursing (e.g., medication management, wound care, medical observations), physiotherapy, rehabilitation, and other health services. Rural municipalities often organize homecare services, nursing homes, assisted living facilities for elderly and people with disabilities, and daycentres for older people in a healthcare center. Urban municipalities mainly organize their home healthcare services with several departments located at their own premises in different areas in the city, but some urban municipalities also organize themselves with both several homecare departments and a healthcare center, with the latter often located in outlying areas.

### Case selection

A case is a homecare unit within the municipality, and each unit is divided into departments. Four Norwegian municipalities were selected based on size, location, and centralization according to the Norwegian Ministry of Local Government [31], not to compare them but to ensure variation of cases providing different nuances and perspectives in the data material. Some municipalities had large numbers of infected citizens and enacted stronger infection control measures. Case selection, therefore, ensures exploration of how municipalities adapt in response to the pandemic (Table 1).

**Table 1:** Case selection and context

Characteristics	Case 1	Case 2	Case 3	Case 4
Municipality description and population	Urban, centralized municipality. 100 000+	Rural, decentralized municipality. 5000–10 000	Rural, centralized municipality. 5000–10 000	Urban, centralized municipality. 100 000+
Organization	Several homecare departments with different bases	Several homecare departments with different bases	One homecare department located in the municipality’s healthcare centre	Several homecare departments with different bases and one department with a base in the local healthcare centre
Number of homecare departments represented	1	2	1	4
Average local infection pressure during the interviews	High	Low	Low	Medium

### Recruitment and participants

A request to participate and an information letter about the study were sent to the top-level manager in each municipality. The manager then recruited other managers throughout the local healthcare system. The inclusion criterion was at least six months of employment in a municipal managerial position during the pandemic. This embedded study identifies four organizational levels and twenty-one managers were recruited to ensure representation of all managerial levels (Table 2): front-line managers with operational and quality responsibility (n = 9); middle managers with departmental responsibilities (n = 5); top-level managers responsible for municipality strategic plans and economy (n = 4); and infection control doctors (n = 3).

**Table 2:** Participants

Participants	Case 1	Case 2	Case 3	Case 4	Total
**Front-line managers**	n = 1	n = 2	n = 1	n = 5	n = 9
**Middle managers**	n = 1	n = 2	n = 1	n = 1	n = 5
**Top-level managers**	n = 1	n = 1	n = 1	n = 1	n = 4
**Infection control doctors**	n = 0	n = 1	n = 1	n = 1	n = 3
**Total**	n = 3	n = 6	n = 4	n = 8	n = 21

### Data collection

We triangulated the data sources from documents and qualitative interviews. The qualitative data consisted of semi-structured interviews. The first author (CS) conducted all interviews, each lasting 30 to 90 min. Two managers agreed to participate only if they could be interviewed together, the other managers were interviewed individually. The interviews were conducted from March to September 2021 using Zoom or Microsoft Teams digital video platforms to comply with strict infection control measures. Written consent was obtained before participation. The interview guide was inspired by Macrae’s [16] Moments of Resilience framework. The framework explores resilience at different scales of activity, to understand how activities interact across levels and how adjustments at the front-line might lead to the reconfiguration of entire systems [16]. The interview covered broad and open questions on several themes: experiences, dialogical practices, and adaptive strategies. The guide was modified for different managerial levels and areas of responsibility, but the content remained the same. To understand the interaction of local (meso-level) and national (macro-level) strategies, the infection control doctors were asked about collaboration with regional and national authorities. The interview guide also included questions about respondents’ demographic characteristics (position, education, job experience). There were 458 pages of transcribed notes from the interviews and all data was encrypted and stored according to UiS guidelines and research approval. The documents included national COVID-19 guidelines and the municipalities’ own contingency plans. These were retrieved by public access to the plans on the municipality’s homepage, and by a request to municipal authorities for preparedness emergency plan.

### Data analysis

**Fig. 1:**
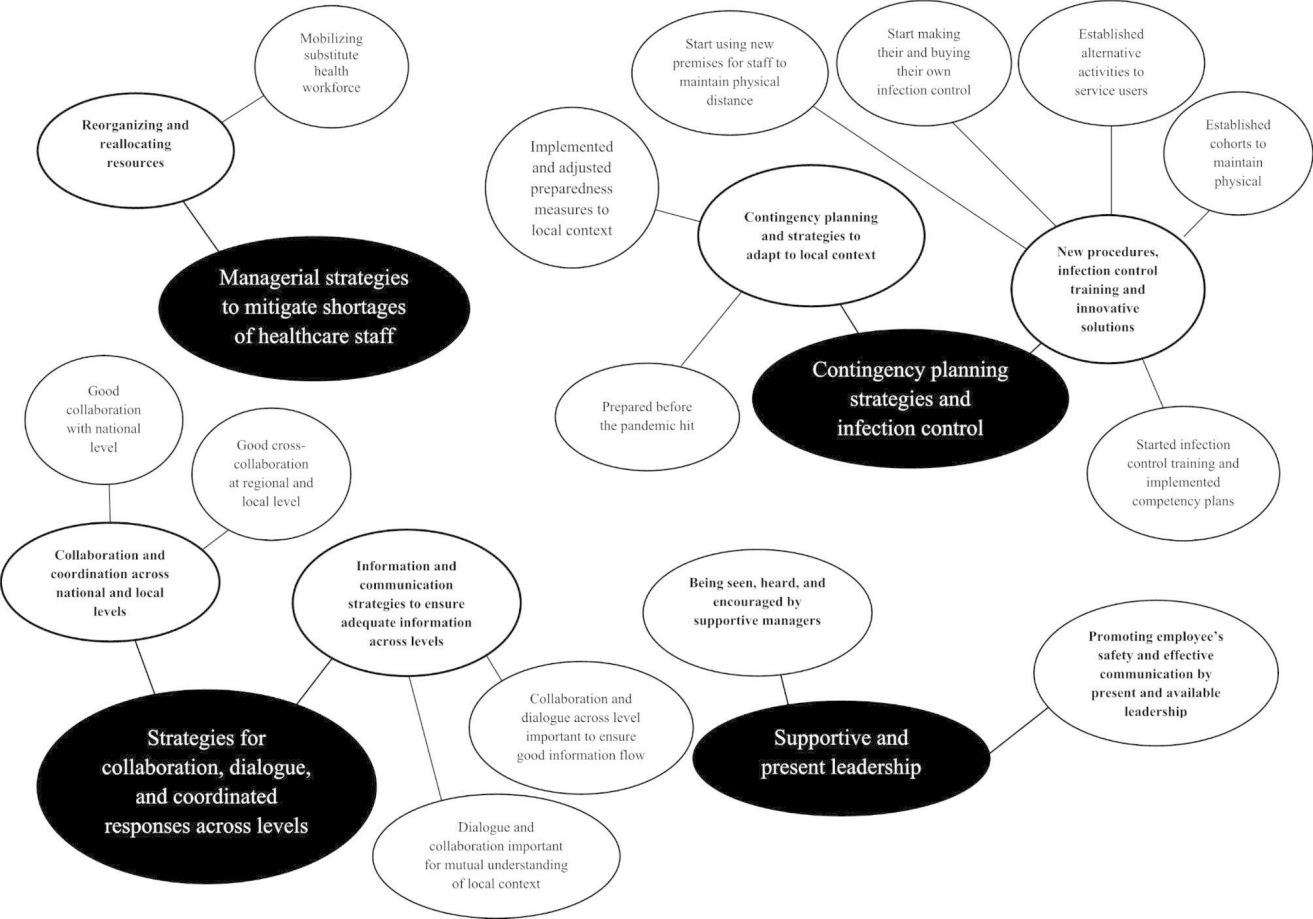
Thematic map

Data from the qualitative interviews was analyzed according to Braun and Clarke’s [32] thematic analysis. An inductive “bottom-up” process led to an understanding of meaning-based patterns within and then across the dataset [32, 33]. The analysis was done by first searching for themes within and then across cases. The first author transcribed the interviews verbatim and uploaded them in NVIVO for analysis. All authors then read the transcripts and the first author highlighted codes evoked from text phrases in the data. In the third phase, these phrases were discussed and arranged into preliminary themes within each case. In the fourth phase, themes were depicted in a visual thematic map suggested by the first author and then reviewed and discussed by all authors to ensure coherence of patterns between the cases. In the fifth phase the themes were revised and renamed, resulting in a final thematic map (Fig. 1). In the sixth phase the results were reported with text extracts from the data. An example from the analysis process shows how themes and sub-themes were identified from data extracts within and across cases (Table 3).

**Table 3:** Example from the analytic process

Cases	Data extract	Coded for	Theme
**Case 1**	*Many of our employees work several different places, so we had to reallocate some of our staff. Some were temporarily employed, and if they worked part-time two different places, they were offered full-time position at one place.*	Reorganizing and reallocating resources	Managerial strategies to mitigate shortages of healthcare staff
**Case 2**	*Then we had to start search for personnel from other parts in the organization and redistribute tasks among staff… … We had some personnel in sick leave that could contribute because their work were facilitated according to what they managed to do.*	Reorganizing and reallocating resources	
**Case 3**	*We implemented new work schedules and cohorts, which cost a lot of money. Many of our healthcare staff works in small positions, so we had to increase their positions in that period.*	Measures to ensure adequate staffing	
**Case 4**	*We have a minimum staffing with two nurses and if one waits for a [Covid-19] test result, we need to take action to ensure proper staffing (…) in case the person doesn’t receive the test result before their shift. So we are ahead*	Hired in extra personnel to ensure adequate staffing	

## Results

The analysis reveals four themes: (1) managerial strategies to mitigate shortages of healthcare staff; (2) contingency planning strategies and infection control; (3) strategies for collaboration, dialogue and coordinated responses across levels; and (4) supportive and present leadership. All managers in the four municipalities described several types of challenges, with different causes (Table 4).

**Table 4:** Challenges

Main Challenges	CASE 1 Urban, centralized municipality. Population: 100 000+ High infection pressure	CASE 2 Rural, decentralized municipality. Population: 5000–10 000 Low infection pressure	CASE 3 Rural, centralized municipality. Population: 5000–10 000 Low infection pressure	CASE 4 Urban, centralized municipality. Population: 100 000+ Medium infection pressure
**Shortage of healthcare staff**	Healthcare personnel working part-time at several employees. Lack of personnel due to quarantine (infection, symptoms, travel and national lock-down). Employees being home with children due to lock down of non-essential services (school, child day care).	Finding resources to new pandemic related tasks (related to geographical location). Balancing resources and competence when reallocating. High absence rate (quarantine) due to travel restrictions (related to geographical location)	Finding resources to new pandemic related tasks. Limited access to resources due to travel restrictions and recommendations of working in one municipality. Balancing resources and competence when reallocating	Lack of personnel due to quarantine (infection, symptoms, travel and national lock-down)
**Lack of preparedness, infection prevention and control**	No practical experience with emergency preparedness and response. Rooms and buildings were not designed in accordance with current infection control measures. Homecare recipients become in need for higher level of care (e.g., nursing home) Lack of infection control equipment	Climatic challenges due to infection control tasks (testing). Long distance between essential healthcare services within the region (e.g., intensive care, PCR-test analysis and transportation). Stressful to handle long term preparedness Home care recipients’ experiences loss of life quality due to lock down of non-essential services. Lack of infection control equipment/date expire on current equipment	Challenging to plan for uncertainty. Unclear roles and responsibility. Challenging to practice user participation. Homecare recipients at nursing home residents became lonely due to lock down of essential services and restrictions due to physical meetings. Lack of understanding due to national infection control measures (low local infection rate). Lack of infection control equipment	Adjusting guidelines, plans and regulations into local context (e.g., the municipality also has a rural part with a healthcare centre). Stressful to handle long term preparedness Homecare recipients experienced loss of health due to lockdown of non-essential services and become in need for higher level of care (e.g., nursing home). Lack of infection control equipment Demanding to handle infection control outbreaks in homecare services. Difficult to split workforce into teams in rural areas
**Information, collaboration, dialogue across units and levels.**	No plans for collaboration across units and departments. Lack of common guidelines for public and private sector	Was not heard and lack of understanding from National Health Authorities due to challenges related to geographical location	Was not consulted and invited to dialogue to discuss local challenges by National Health Authorities in an early phase in the pandemic. Challenging to be a part of a large region due to local differences and challenges	Constantly changing national guidelines led to challenges with information flow across levels. Digital platforms not suitable for dialogue in groups
**Remote leadership**	Home office and remote leadership. High workload for managers	Lack of knowledge about nursing leadership (managers with other types of education)	Remote leadership made it challenging to interact with employees and colleagues	Managers had high workload. Remote leadership made it difficult to support employees

### Managerial strategies to mitigate shortages of healthcare staff

To compensate for staffing challenges caused by COVID-19, the managers used strategies such as reorganization and reallocation of staff (e.g., physiotherapist, occupational therapists’ staff from daycentres and cafeterias) from units, departments, and services that had been closed or cut back during the lockdown. To ensure adequate resources in homecare services, managers offered their part-time workers temporary full-time positions to prevent the spread of infection from workplaces to employees, and homecare recipients. The contextual conditions were different for Case 3 where many part-time workers and substitutes had their main position outside the municipality or in other countries. Therefore, they increased the number of staff on each shift and changed the work schedule, so the staff worked more and longer shifts. Managers also mapped homecare recipients who were not in urgent need of healthcare services, and by reducing non-essential services they lightened the workload of personnel. While some employees offered to take on extra work or double shifts, new tasks created by the pandemic forced managers to ensure that the right skills were meeting the right needs. Case 2 differentiated from the other cases as they were imposed new pandemic-related tasks because of its geographic location. Sick listed employees in need for facilitated work contributed with tasks they were still able to perform, in that way managers ensured sufficient resources to these new assignments.

During the pandemic, managers designed and introduced new work schedules to ensure adequate staffing. Managers in Case 1 established a substitute health workforce team that could step in if the absentee rate became too high. They also agreed to reallocate staff to departments with high infection pressure that were under-resourced. In Case 2, managers made arrangements with part time employees that their position could be quickly increased in an alternative work schedule as a rapid response to acute staffing shortages. One department in Case 4 did not have any trouble finding resources as everyone offered to work more than usual, in addition to medical and nurse students being home from school due to restrictions of physical distance. However, the manager was proactive and hired extra personnel in case of absence, ensuring adequate coverage:

*“We have a minimum staffing with two nurses and if one waits for a test result, we need to take action to ensure proper staffing for that weekend in case the person doesn’t receive the test result before their shift, so we are ahead.”* (Front-line manager, Case 4).

### Contingency planning strategies and infection control

#### Contingency planning and strategies to adapt to local context

Preparedness plans were found insufficient as they did not plan for a long-term pandemic. A top-level manager in Case 1 stated that the managers had no practical experience with emergency preparedness work. So, while managers waited for national guidelines, the municipalities took the initiative. By keeping themselves apprised of the pandemic through media and international healthcare organizations, infection control doctors started to prepare to treat outbreaks in their municipalities. Managers developed several types of plans and routines in preparation for a variety of scenarios. They made plans to respond to possible challenges to the healthcare services within their community. One infection control doctor in Case 3 explained how he started preparing the municipality for the first pandemic outbreak by calculating the number of hospital beds and infection control equipment they would need based on the infection rates reported from Italy. Some municipalities implemented targeted measures to respond rapidly to changes in the local infection status. In Case 1 the municipalities introduced contingency plans for staff shortages and homecare services and nursing homes were told to plan for a 30–40% staff reduction. Case 4 planned for the types of services they could cut back if there were staffing shortages resulting from infection or quarantine, and still safely deliver patient care. The municipality established committees with responsibility for different areas under pandemic management, which gave the infection control doctor in Case 4 the experience of the decision-making process becoming more solid. They also applied an improvement tool which made it possible to evaluate and adjust their strategies, to improve their handling of the pandemic. Managers in Case 2 emphasized the helpfulness of knowledge and experience from previous crises. They started assessing the risk of national and local scenarios and what it would entail for the different services.

Outdoor testing stations were used to test as many people as possible while protecting them from infection. However, due to Norway’s cold climate, managers in Case 2 had to find alternatives:

*“We need proper conditions for testing, so we had to rent suitable premises nearby. Because after we had premises for testing, it became too cold in there and the antigen rapid test could be temporarily affected. So, we needed to do some adjustments along the way, because it can easily be too cold. We wanted an outdoor test station so people didn’t need to leave their cars as we could see many other places, but then people had to work in an outfit used for snowmobile driving, so we couldn’t do it outdoors.”* (Infection control doctor, Case 2).

#### New procedures, infection control training and innovative solutions

To prevent outbreaks, managers devised new routines to ensure physical distancing and a handling of infection among homecare recipients. All managers divided their teams into smaller groups and established new premises to reduce the number of contacts and increase physical distance. To ensure infection control and limit the spread of infection in healthcare institutions, one municipality remodelled its nursing homes to meet the latest infection control standards. Knowledge of infection control was quickly identified as a field in which employees needed to be trained, and new routines for infection control were introduced. Types of training varied but dress rehearsals for personal protective equipment (PPE) were common. Managers in Case 1 required all nursing students to be trained in infection control before beginning their training in homecare. Managers also hung posters and placed infection control videos on digital platforms. In Case 1, managers offered digital infection control courses:

*“We used the quarantine period actively for courses and training…. So many needed infection control training and while they were home in quarantine they might as well participate in the training courses.”* (Middle manager, Case 1).

To handle infection control outbreaks in homecare services, the managers appointed specialized teams and nurses with in-depth knowledge in infection control, so they could quickly take control of outbreaks. In Case 2, suitable premises were established for teams serving homecare recipients infected with COVID-19 to minimize the chances of spreading infection to the staff.

To handle PPE shortages, the managers contacted stores, educational institutions, and individuals with 3D-printers with requests to make protective goggles and face shields. When daycentres for people with dementia were shut down, many of these people lost their only opportunities for social interactions or physical activity. All municipalities then offered alternative solutions such as outdoor concerts and physical activities. In Case 3, the music therapist working at the daycentre helped teach the users to learn digital platforms and played the accordion for them on a YouTube channel.

### Strategies for collaboration, dialogue, and coordinated responses across levels

#### Collaboration and coordination across national and local levels

The managers in the four municipalities had different perceptions of collaboration and dialogue between the national and local levels. The three most centralized municipalities, regardless of population, reported being heard, involved, and invited to provide feedback as a part of the development of new national guidelines that would ensure a mutual handling of the pandemic. However, for managers in Case 2, decentralization and location posed challenges due to insufficient dialogue with national authorities. They highlighted collaboration and dialogue with the county governor since they had a mutual understanding of local conditions and challenges. Regional collaboration was also cited as important by the infection control doctors in Case 3 and Case 4, as a measure to share knowledge and reduce the regional impact of infection. They met with infection control doctors, the regional hospital and the county governor to keep each other apprised of infections in the region and coordinate responses to outbreaks.

Collaboration among managers with different responsibilities led to development of new plans and routines. For example, in Case 3 they established an infection control forum with representatives both from the homecare services and other healthcare institutions within the municipality, including managers, school nurse and the public health coordinator. In this forum they agreed upon a course of action. In Case 1, there was a lack of collaborative plans and systems across the public and private sectors. In response, healthcare managers enabled cross-sectoral collaboration by sharing a mutual quality system in which all sectors complied with municipal guidelines. Managers in Case 2 reallocated resources to new pandemic-related tasks:

*“… you do not have to be a healthcare professional to do everything. The infection tracking team … we managed to train people from the personnel, technical and the financial department to do that. People that had work tasks that could be set aside if we needed them. So, we have improved our cross-sectoral collaboration. (…) We might be too focused of working within our respective sector, we need to be able to utilize our resources in a better way.”* (Middle manager, Case 2).

#### Information and communication strategies to ensure adequate information across levels

Managers mentioned challenges with information flow as new guidelines were constantly changing, sometimes daily. To ensure that all managers received sufficient information at all times, digital meetings became important. Managers in some the municipalities talked about a coronavirus hotline that they could call with questions or if they needed information on the pandemic. The managers ensured that staff had up-to-date information at all times.

Workplace by Facebook, a business version of Facebook, was usually applied but managers also used information screens, posters, e-mail, and SMS. Several managers had to interpret the information and guidelines from national authorities, tailor them to the local context and make it comprehensible for staff. One manager described the importance of communicating information in a way that subordinates would understand:

*“I think it was a challenge to interpret when you got these restrictions and recommendations, right? Interpreting them into a local context requires you to be clear. Like I am clear giving information to my managers, and it was not always that I had the information I needed to be clear enough.”* (Middle manager, Case 2).

### Supportive and present leadership

#### Promoting employees’ safety and effective communication by present and available leadership

All managers commented on the difficulty of remote leadership, especially at the front-lines where managers emphasized the importance of being present and not letting employees feel abandoned. Fear of infection was common among personnel. By being present, managers were able to see their employees’ struggles, communicate with them, support them and making sure they felt safe. Front-line managers insisted on the importance of being physically present at their unit instead of working from a home office. By presence, front-line managers found it easier to be involved in everyday work, to have a sense of the situation, ensure that all staff had the information they needed and solve problems as they arose. Middle and top-level managers found it important to be available for their subordinates. One top-level manager in Case 4 explained communicating to the middle managers that they could call anytime, and that being available created an environment of psychological safety in a period of uncertainty. In situations where the workload was heavy, managers were physically present. In the words of one middle manager:

*“Many of our staff are tired after last Christmas. There was an infection outbreak in our nursing home and the front-line manager worked double shifts almost the entire period, and you see what that meant for her staff. There were hardly any nurses left, so she had to work as a nurse and that meant a lot. And what she established by doing that, that’s valuable. She formed bonds with her employees which I think is very important for future work.”* (Middle manager, Case 3).

#### Being seen, heard, and encouraged by supportive managers

All managers talked about an increasingly heavy workload during the pandemic. One front-line manager noted that being heard by the middle manager made it easier to cope with the high workload. Front-line managers also made their staff feeling appreciated praising their hard work and complimenting them for handling difficult situations well. Several front-line managers highlighted that having a good work environment made it easier to cope with the situation. A middle manager mentioned the importance of having support from above when decisions were made. Most of the collaboration between middle and top-level managers took place on digital platforms, and strategies to see and follow up with subordinate managers was to check on them regularly. According to a top-level manager:

*“I think I’ve been a little more focused on each and every manager and to see them every day. It’s been a lot of … we`ve had home office and everything happens digitally, so it has affected the interaction I’ve had with my managers below me. But I’ve been good at calling them on Skype just asking them how they are today. (…) So, it’s been a way of seeing them in everyday work.”* (Top-level manager, Case 2).

## Discussion

The purpose of this study was to examine which strategies managers in homecare services apply when adapting to the challenges induced by the COVID-19 pandemic. We also wanted to shed light on the ways in which contextual factors might influence managers’ responses to uncertainty. Although the managers in all four municipalities reported similar challenges, contextual variations affected their experience of challenges and their choice of strategies. We discuss the results in relation to previous research on resilience in healthcare theory.

### The role of managerial strategies in supporting resilience in healthcare

**Fig. 2:**
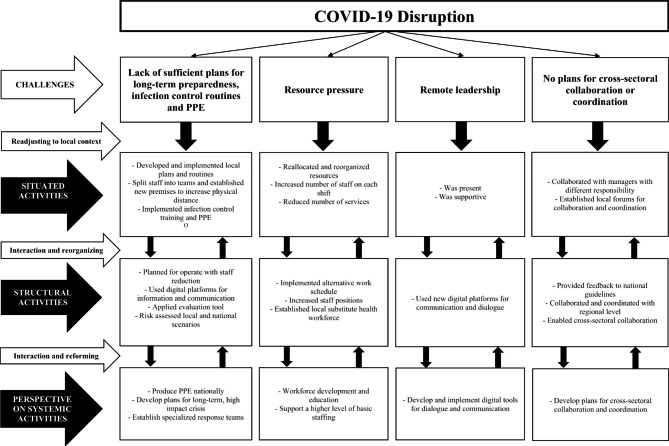
Managerial strategies within different moments of resilience

The results of this study are consistent with the findings in a recent paper suggesting that managers contribute to organizational resilience by making trade-offs, adjustments, and adaptations [18]. Macrae [16] suggests that resilience unfolds at three scales of organizational activity: situated, structural and systemic moments of resilience. This study shows how healthcare managers adapted to the pandemic by using strategies within situated and structural moments of resilience. These moments might trigger systemic resilience, leading to a reconfiguration of the system and the reorganization of homecare services during a crisis (Fig. 2). Situated resilience occurred when managers found effective strategies to compensate for insufficient plans and lack of healthcare professionals in the first outbreak of the pandemic. Reallocating and reorganizing resources between sectors and institutions in addition to implementing alternative work schedules were creative solutions to understaffing. These results are similar to a single case study in Norway exploring front-line and middle-managers’ strategies during the pandemic [9].

Consistent with previous studies [9, 34, 35] the results here showed the importance of supportive and present leadership during the pandemic. Front-line managers emphasized the need to be physically available to their staff as they perceived they were able to influence the psychological environment in a positive way. This corresponds to previous research which describes present leadership as important for a trust-based relationship between managers and their staff, and to ensure patients’ safety [11]. Supportive and present leadership should be highlighted within management crisis education and training, as an important strategy when responding to healthcare crises such as pandemics.

In this study, the managers’ strategies ranged from situated resilience activities by being available and supportive whenever staff needed them, to structural resilience activities by developing creative solutions to handle the pandemic and to recognize individual needs.

Strategies to ensure infection control training and to distribute personal protective equipment (PPE) were used in preventing the spread of contagion between service users and healthcare staff in homecare services. These strategies were initially rapid responses to a major crisis but evolved into more structural activities as managers learned how to create sustainable work practices. Examples were training programs, infection control protocols, specialized teams and communication that ensured that everyone had the information to prevent or contain potential outbreaks. These strategies facilitated dynamic collaborative learning and knowledge-sharing which is fundamental to developing new work practices and responding to uncertainty by improving quality [12, 35]. In addition, by enabling a cross-sectoral and multi-level collaboration within the local health system, the managers were able to find and utilize resources outside the healthcare sector, which is an important strategy to improve quality [15].

These structural resilience activities enabled managers to share knowledge and information and reduce the impact of the virus. These results are in line with previous studies showing that cross-level collaboration led to the allocation of scarce resources, preparedness for planned changes and interventions, and has been an important factor for learning during daily activity [18, 36, 37]. Collaboration and coordination across sectors and levels are highlighted as important resilience strategies [38] and was also important for learning and solving staffing challenges in different healthcare settings during the pandemic [10, 39]. Knowledge of how effective coordination, dialogue and collaboration across sectors contributed to new solutions, rapid decision-making and learning might be a key lesson for handling future pandemics.

Monitoring and learning can improve system performance [40]. By continuously evaluating their own performance, managers could learn and modify their handling along the way. Evaluating and learning from the early phases of the pandemic, was also important to hospital managers in the United States as they prepared for the next wave [34].

### The role of local context

Geographical location was important for what strategies managers used and how they adapted to the challenges posed by the pandemic. The significance of contextual factors in managers’ quality work and their adaptation to different situations are in line with previous studies [35, 37, 41]. Balancing capacity (e.g., resources, competence) and external demands (e.g., laws, guidelines, regulatory demands) have been highlighted as difficult in decentralized areas [23]. Recruiting and maintaining a healthcare workforce has been identified to be interrelated with rural associated factors [42]. However, this study adds to the knowledge of how geographical location influences homecare managers’ response to a crisis. The lack of qualified healthcare personnel even before the pandemic hit, and managers’ reliance on outside resources made the increased number of tasks imposed by the government an additional burden. However, they were creative enough to look beyond traditional solutions and engage in cross-sectoral recruitment in addition to facilitate work for sick listed employees. These strategies, in addition to implementing an alternative work schedule, made it possible to handle staff shortages despite high demand, and are examples of structural resilience activities (Fig. 2). Healthcare systems’ capacity to adapt to local context, despite stress and resource pressure is essential to maintain normal functioning and build resilient healthcare organizations [38].

Rural and decentralized areas tend to be more vulnerable to infection outbreaks, due to limited access to essential healthcare services (e.g., specialist healthcare) and the limited number of hospital beds [21]. They should therefore prepare to prevent outbreaks. Awareness of their own vulnerability, managers in decentralized areas prepared by conducting national and local risk assessments and prepared local targeted measures at each level. This strategy might be a result of lessons learned from previous crisis [43] and demonstrates that successful adaptations promote learning and development [18] and that adaptations is driven by the opportunities to develop innovative solutions that increase the system’s efficiency [14].

In the first phase of COVID-19 in Norway, it was difficult to apply contingency plans due to uncertainty arising from the lack of crisis scenarios accounting for their asymmetric impact (e.g., vulnerable groups, geographical challenges). Echoing other studies [44], our study found that the government’s constantly changing guidelines and measures were not tailored to all local contexts and imposed more challenges than solutions. Hence, by learning from the COVID − 19 pandemic, policy makers should allow for the municipalities to implement targeted measures adjusted at a local level to minimize the impact of and quickly respond to challenges that may arise in future pandemics.

The document review revealed that national and municipality preparedness plans were outdated and failed to account for a long-term pandemic with such a cross-sectoral impact. This finding is consistent with reports from the early phase on the pandemic, both from Norway [2, 9, 45] and abroad [46]. By constantly revising guidelines and procedures for their local health system, and by disseminating information and training staff in new procedures, managers improved their understanding of current practice and enforcement. This is in line with managers’ previous descriptions of how clear communication to their staff contributed to understanding, sense-making and preparedness for new situations [18, 47].

According to a recent review, managers who demonstrated good communication despite being under chronic stress was a crucial capacity to strengthen resilience [38]. The ability to adapt rapidly to the constantly changing information and circumstances and to ensure adequate and timely information dissemination was also identified as the key lessons from the SARS epidemic [7].

### Strengths and limitations of the study

This study has several strengths and limitations. To our knowledge, this is the first study to explore managers’ strategies and the role of contextual factors in handling the pandemic in Norwegian homecare services. This is valuable knowledge for handling planned changes and crises in healthcare. A larger and more geographically diverse sample of municipalities could have added richness to our study. However, the sample size provided rich information, and the results are relevant and might be transferable to similar settings. The variations in size, geographical location, and infection pressure during data collection have contributed to a broad variation in challenges and strategies during different phases of the pandemic.

The interviews were conducted 12–18 months after the pandemic was declared, so valuable information about the early phase experiences and handling might have been missed due to possible recall bias among the participants. However, the pandemic was still ongoing when the interviews were conducted, and we experienced that the participants shared detailed experiences and examples of strategies used during the pandemic, including the first phase.

## Conclusion

In this paper we found that home care managers responded quickly to COVID-19 induced challenges. Multi-level adaptations to accommodate local challenges and needs in home healthcare services were essential to handling the COVID-19 pandemic in Norway. Following our results, future national preparedness plans and strategies need to be more flexible in terms of allowing for adaptations to local context and provide managers room for making necessary adjustments and adaptations in their organization. Effective and targeted responses to a crisis requires strategies that promote collaboration and coordinated responses throughout a healthcare organization. This study also illuminates the importance of interaction between scales of activity. We therefore suggest a new theoretical contribution to understand managers’ capacity to adapt at different system levels (Fig. 2). Recommendations for future research would be a longitudinal exploration of managers’ experiences and strategies from a micro and macro-level perspective. Such a study would provide valuable information on interactions across all levels and a deeper insight into managers’ handling of long-term crisis in healthcare. Such an approach could strengthen theoretical frameworks by deepening our understanding of managers’ role in resilient healthcare.

## Data Availability

Data from the interviews are available upon reasonable request to the corresponding author.

## Declarations

The author confirms that all methods were performed in accordance with regulations and guidelines.

## Abbreviations

SSB: Statistics Norway
PPE: Personal Protective Equipment
